# Strategies for the management and prevention of withdrawal syndrome in critically ill pediatric patients: a systematic review

**DOI:** 10.5935/0103-507X.20220145-en

**Published:** 2022

**Authors:** Kassiely Klein, Jéssica Silveira Pereira, Kátia Adriana Lins Jaines Curtinaz, Leonardo Bigolin Jantsch, Neila Santini de Souza, Paulo Roberto Antonaccio Carvalho

**Affiliations:** 1 Postgraduate Program in Child and Adolescent Health, Faculdade de Medicina, Universidade Federal do Rio Grande do Sul - Porto Alegre (RS), Brazil.; 2 Postgraduate Program in Pathology, Universidade Federal de Ciências da Saúde de Porto Alegre - Porto Alegre (RS), Brazil.; 3 Department of Health Sciences, Universidade Federal de Santa Maria, Campus Palmeira das Missões - Palmeira das Missões (RS), Brazil.; 4 Department of Pediatrics, Faculdade de Medicina, Universidade Federal do Rio Grande do Sul - Porto Alegre (RS), Brazil.

**Keywords:** Substance withdrawal syndrome, Analgesics, opioid, Hypnotics and sedatives, Intensive care units, pediatric

## Abstract

**Objective:**

To verify strategies for the prevention and treatment of abstinence syndrome in a pediatric intensive care unit.

**Methods:**

This is a systematic review in the PubMed database^®^, Lilacs, Embase, Web of Science, Cochrane, Cinahl, Cochrane Database Systematic Review and CENTRAL. A three-step search strategy was used for this review, and the protocol was approved in PROSPERO (CRD42021274670).

**Results:**

Twelve articles were included in the analysis. There was great heterogeneity among the studies included, especially regarding the therapeutic regimens used for sedation and analgesia. Midazolam doses ranged from 0.05mg/kg/hour to 0.3mg/kg/hour. Morphine also varied considerably, from 10mcg/kg/hour to 30mcg/kg/hour, between studies. Among the 12 selected studies, the most commonly used scale for the identification of withdrawal symptoms was the Sophia Observational Withdrawal Symptoms Scale. In three studies, there was a statistically significant difference in the prevention and management of the withdrawal syndrome due to the implementation of different protocols (p < 0.01 and p < 0.001).

**Conclusion:**

There was great variation in the sedoanalgesia regimen used by the studies and the method of weaning and evaluation of withdrawal syndrome. More studies are needed to provide more robust evidence about the most appropriate treatment for the prevention and reduction of withdrawal signs and symptoms in critically ill children.

**PROSPERO register:**

CRD 42021274670

## INTRODUCTION

An increasing number of patients admitted to the pediatric intensive care unit (ICU) are subjected to the use of sedatives and analgesics. Sedatives aim to reduce anxiety and agitation caused by the environment, maintain invasive methods and devices, and optimize mechanical ventilation (MV). In turn, analgesics are intended to minimize and/or eliminate pain caused by the disease itself and by performing procedures.^(1-3)^

Opioids and benzodiazepines are often present in pediatric intensive care, but prolonged use can trigger unwanted side effects, such as withdrawal syndrome. Withdrawal syndrome has been recognized since the 1990s and is characterized by autonomic dysregulation, central nervous system excitation and gastrointestinal symptoms that occur after the reduction or abrupt interruption of the infusion of sedative analgesic drugs, usually within the first 24 hours; the condition may improve when there is a return of its administration or the use of other appropriate drugs.^(4,5)^ Critically ill patients who receive high doses or are exposed to opioids and/or benzodiazepines for more than 72 hours are at high risk of developing withdrawal syndrome.

In the current literature, abstinence syndrome has a high incidence rate, approximately 64.6% in pediatric patients, and this may be associated with the absence of standardized definitions and measures in the diagnosis of the withdrawal syndrome, the inconsistent weaning of opioids and/or benzodiazepines between studies, the performance of the study in different populations and the lack of protocols regarding the dosage, administration and weaning of sedoanalgesia, which prevents the homogeneity of studies.^(6,7)^

It is observed that the basis of treatment for withdrawal syndrome is gradual weaning, and it is extremely important to recognize the signs and symptoms of withdrawal and perform management with rescue therapies, in which continuous short-acting infusions are replaced with sedative agents and long-acting analgesics, preferably in the enteral presentation, and short-acting drugs should only be used as rescue therapy when acute withdrawal symptoms appear.^(8)^

Currently, the drugs most often used for weaning from sedoanalgesia are enteral methadone and morphine in the opiate group, lorazepam and clorazepate in the benzodiazepine group, and alpha-2 agonists such as clonidine and dexmedetomidine. A study that recognized the weaning profile of a pediatric ICU in Brazil showed that the most administered drugs were lorazepam, methadone and clonidine in 41.5% of patients. ^(9,10)^

Even so, there is a large gap in the evidence regarding the use of these drugs for the treatment of withdrawal syndrome; there is conflict and concern about the safety of using long-acting enteric agents, in addition to great differences regarding dosages and administration intervals.^(11-14)^

There are validated scales for the evaluation and recognition of the signs and symptoms of withdrawal syndrome, such as the Sophia Observation Withdrawal Symptoms Scale (SOS), the Withdrawal Assessment Treatment (T-1) and the Finnegan scale. However, withdrawal syndrome is still underreported and can be easily confused with other clinical conditions, as its signs and symptoms are highly variable and can be affected by age, medical condition, exposure time and type of drug used.^(7)^

Thus, there is a need and interest in verifying, in the national and international literature, the existing studies on the treatment and prevention of withdrawal syndrome in pediatric ICUs. There is no gold standard and a great difference of opinion as to which drugs to use and in what dosages, as well as strategies to be used in the treatment and prevention of withdrawal syndrome. Thus, this study aimed to verify, through a systematic review, strategies for the prevention and treatment of withdrawal syndrome in pediatric ICUs.

## METHODS

This is a systematic review conducted according to the Preferred Reporting Items for Systematic Reviews and Meta-Analyses (PRISMA) recommendations.^(15)^ and the Cochrane Handbook.^(16)^ This systematic review was registered and approved in PROSPERO under the CRD protocol. 42021274670.

### Definition of the research question

The research question was developed using the PICOS strategy, and the population was (P) critically ill pediatric patients; intervention (I) measures to prevent and reduce symptoms; comparison (C) of types of treatment or interventions; outcome (O) of withdrawal syndrome; and study designs (S) were observational or experimental. Thus, the following question was asked: “What are the most often indicated measures to prevent and reduce the symptoms of withdrawal syndrome in critically ill children?”

### Search strategy

Searches were performed in the databases PubMed^®^, Latin American and Caribbean Health Sciences Literature (Lilacs) of the Virtual Health Library (VHL), Embase, Web of Science, Cummulative Index to Nursing and Allied Health Literature (Cinahl), Cochrane Database Systematic Review (CDSR) and CENTRAL. A three-step search strategy was used for this review.

An initial search was limited to MEDLINE^®^ (PubMed). This method is used to better understand the subject and identify other relevant terms. This allows the development of an initial search strategy, which identifies additional terms and excludes nonrelevant terms (Table 1). After choosing the appropriate terms, translation into the other databases of interest was performed.

**Table 1:** Database search strategy via PUBMED

Consultation	Mapping of terms	Retrieved records
1#	“Substance Withdrawal Syndrome/therapy”[mh] OR “Substance Withdrawal Syndrome/prevention and control”[mh] OR withdraw^*^[tw] OR Abstinen^*^[tw]) AND	
2#	“Iatrogenic Disease/therapy”[mh] OR “Iatrogenic Disease/prevention and control”[mh] OR “Analgesics, Opioid/therapeutic use”[mh] OR “Benzodiazepines/therapeutic use”[mh] OR “Morphine Derivatives/therapeutic use”[mh] OR “Fentanyl/therapeutic use”[mh] OR “Dexmedetomidine/therapeutic use”[mh] OR “Ketamine/therapeutic use”[mh] OR “Iatrogen”^*^[tw] OR “Hospital-Acquired”[tw] OR “Opioid^*^[tw] OR “Benzodiazepin”^*^[tw] OR “Morphine”[tw] OR “Codeine”[tw] OR “Hydrocodone”[tw] OR “Oxycodone”[tw] OR “Dihydromorphine”[tw] OR “Ethylmorphine”[tw] OR “Hydromorphone”[tw] OR “Oxymorphone”[tw] OR “Thebaine”[tw] OR “Phentanyl”[tw] OR “Fentanyl”[tw] OR “Alfentanil”[tw] OR “Sufentanil”tw] OR “Midazolam”[tw] OR “Dexmedetomidine”[tw] OR “Ketamine”[tw]) AND	437 results
3#	“Critical Illness”[mh] OR “Critical Care”[mh] OR “Intensive Care Units, Pediatric”[mh] OR “Critical Illness”^*^[tw] OR “Critically Ill”[tw] OR “Critical Care”[tw] OR “Intensive Care”[tw] OR “ICU”[tw] OR “NICU”[tw] OR “PICU”[tw]) AND	
4#	“Child”[mh] OR “Infant”[mh] OR “Child”^*^[tw] OR “Preschool”^*^[tw] OR “School”^*^[tw] OR “Infant”^*^[tw] OR “Newborn”^*^[tw] OR Neonat^*^[tw] OR “Paediatric”^*^[tw] OR “Pediatric”^*^[tw])	

Data collection took place on July 19, 2021, using the “advanced search” feature with the descriptors Medical Subject Headings (MeSH) and Boolean operators “ OR ” and “AND”. The searches were performed by two independent examiners in July 2021, strictly complying with the preestablished methodology. The searches were delimited from 2010 onward to focus this study on the current literature.

### Inclusion criteria

Inclusion criteria were as follows: studies evaluating pediatric patients aged > 28 days and < 21 years, using sedoanalgesia, and aiming to identify strategies for the treatment, reduction and prevention of withdrawal syndrome were included. Original studies of randomized controlled trials (RCTs) and non-randomized clinical trials (NRCTs) available in Portuguese, English and/or Spanish, which had full text available, were also eligible. No restrictions were imposed regarding the study design, thus including observational and experimental studies.

### Exclusion criteria

Exclusion criteria were as follows: literature reviews that addressed the treatment of childhood withdrawal syndrome at home, studies with adult populations or exclusively neonatal populations, and incomplete studies or studies with data not published in full. Studies with a retrospective design and a sample size < 50 were also excluded because they had lower methodological quality and a likelihood of research bias. Finally, studies published before 2010, conference abstracts or articles retracted due to data fraud were also excluded.

### Data extraction

Initially, the records were exported to the Zotero reference management *software* version 5.0. Two review authors independently conducted the initial evaluation of the relevant records after excluding duplicate articles. Researchers began the selection process by reading the titles, abstracts and, finally, the full text. Based on this, a collection of studies was created to be evaluated by the reviewers. Differences in selection were resolved by consensus and/or a third reviewer.

**Figure 1:**
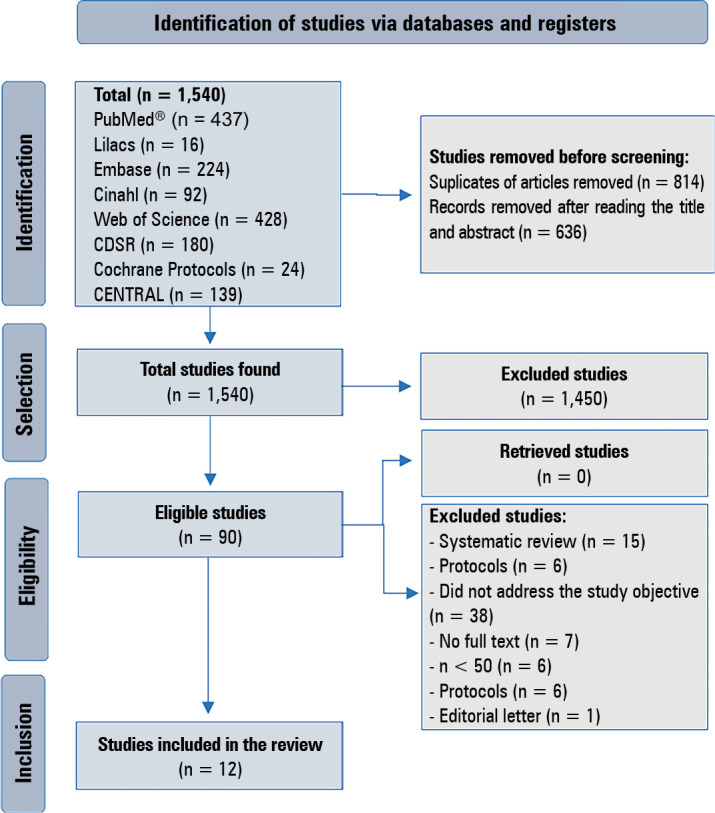
Selection of studies.

The data were extracted and compiled in an Excel spreadsheet, version 16.0 (Microsoft®). The spreadsheet contained the following data: study identification, title, journal, authors, year of publication, country of study, study design, age of the population, sample size, inclusion and exclusion criteria, instrument for identifying the withdrawal syndrome, description of methods for the prevention and treatment of withdrawal syndrome, incidence of withdrawal syndrome and outcome. After data collection, the information was tabulated with subsequent analysis, interpretation and preparation of the study. The results of the selection are presented in a flowchart of PRISMA items (Figure 1).

Lilacs - Latin American and Caribbean Literature on Health Sciences; Cinahl - Cumulative Index to Nursing and Allied Health Literature; CDSR - Cochrane Database Systematic Review.

### Risk assessment and bias

The evaluation of methodological quality was performed by two researchers. The clinical and crossover studies were evaluated using the Revised Cochrane Risk-of-Bias Tool for Randomized Trials (RoB 2.0) to assess the risk of bias in RCTs, the Risk Of Bias In Non-randomized Studies of Interventions (Robins-I) for NRCT and the Joanna Briggs Institute (JBI) risk assessment list. To assess the risk of bias in RCTs, RoB 2.0 is currently the tool recommended by the Cochrane collaboration. According to the tool, for each study result of interest, five domains are evaluated regarding possible study biases. The five domains are as follows: bias in the randomization process, deviations from the intended intervention, bias due to missing data, bias in the measurement of outcomes and bias in the reporting of outcomes.^(17)^

Robins-I, a tool also produced by Cochrane, seeks to assess the risk of bias in the results of non-randomized studies that compare the health effects of two or more interventions.^(18)^

For observational cohort studies, the JBI checklist of cohort studies, which evaluates the methodological quality of a study, was used; this checklist determines whether a study addressed the possibility of bias in its design, conduct and analysis. It consists of 11 items, which are scored as “yes”, “no”, “unclear” or “not applicable”.

## RESULTS

The search strategy found 1,540 studies, of which 814 were removed because they were duplicates and 636 after reading the titles and abstracts because they did not fit the objective of the study. Ninety articles were analyzed in full, leaving 12 that met the eligibility criteria and were included in this systematic review (Figure 1).

### Characteristics of the studies

Of the 12 selected studies, four were RCTs;^(19-22)^ three were NRCTs;^(8,23,24)^ and five were observational studies.^(25-29)^ All studies were conducted in pediatric ICUs. The country that conducted the most research on the subject was the United States with four.^(8,19,24,28)^

Our 12 studies enrolled a total of 1,273 individuals. The age of the selected patients ranged from zero to 21 years of age (Table 2).

**Table 2:** Characteristics of the selected studies

Author, country	Methodology	Population	n	Age	Control/Intervention
Amirnovin et al.,^(8)^ United States	Foresight before and after intervention	Children admitted to the pediatric cardiac ICU who received opioid infusions ≥7 days	119	< 21 years (mean 10 months)	Control: weaning at medical discretion Intervention: protocolized weaning
Bowens et al.,^(19)^ United States	Prospective, double-blind, randomized	Children admitted to the pediatric ICU with ≥ 5 days of fentanyl infusion	68	> 28 days to < 18 years (mean 4.4 months)	Control: protocolized management of WS in “low doses” (according to weight) of methadone Intervention: protocolized management of WS using “high-dose” methadone (according to fentanyl infusion rate)
Garisto et al.,^(20)^ Italy	Randomized clinical trial	Children admitted to the pediatric ICU with congenital heart disease	48	> 28 days to < 24 months (mean 5.5 months)	Control: use of opioids and benzodiazepines alone Intervention: use of benzodiazepines and opioids with dexmedetomidine
Hünseler et al.,^(21)^ Germany	Prospective, double-blind, randomized controlled trial	Children admitted to the pediatric ICU on MV for more than 3 days and on midazolam and fentanyl	219	NB with GA > 37 weeks up to 2 years (mean 10 months)	Control: patients received clonidine infusion Intervention: patients received a placebo infusion
Tiacharoen et al.,^(22)^ Thailand	Open, randomized and controlled study	Children who received intravenous sedatives or analgesics for ≥ 5 days	30	> 1 month and < 18 years (mean 20.76 months)	Control: weaning at medical discretion Intervention: weaning was protocolized through risk assessment for the development of WS
Gaillard-Le Roux et al.,^(23)^ França	Prospective, before and after	Children admitted to the pediatric ICU	194	> 28 days to < 18 years (mean 6.6 months)	Control: weaning at medical discretion
Sanchez-Pinto et al.,^(24)^ Estados Unidos	Prospective pre- and post-intervention	Children admitted to the pediatric ICU who received scheduled opioids for ≥ 7 days	107	< 21 years (mean 26.4 months)	Control: weaning at medical discretion Intervention: protocolized weaning
Geven et al.,^(25)^ Holanda	Retrospective observational	Children admitted to the pediatric ICU who used benzodiazepines and opioids for 48 hours continuously	102	< 18 years (mean 14 months)	Observation of patients weaned on dexmedetomidine after use of benzodiazepines and opioids
Sperotto F, et al.,^(26)^ Italia	Observational prospective	Patients < 18 years of age who received dexmedetomidine for a period greater than or equal to 24 hours	163	< 18 years (mean 13 months)	Observation of patients before and after 24 hours of dexmedetomidine infusion
van der Vossen et al.,^(27)^ Holanda	Retrospective cohort	Children admitted to the pediatric ICU	73	< 18 years (mean 63.3 months)	Control: evaluation of patients before conversion from midazolam to lorazepam Intervention: evaluation of patients 48 hours after conversion
Sanavia et al,^(28)^ Espanha	Observational prospective	Children admitted to the pediatric ICU who received continuous infusions of sedatives and analgesics for > 4 days	100	> 1 month to 16 years (mean 8 months)	Observation of patients using medication rotation protocol
Berrens et al.,^(29)^ Estados Unidos	Retrospective study	Children admitted to the pediatric ICU	50	> 1 month to < 18 years (mean 24 months)	Observation of patients weaned on clonidine compared to patients weaned on dexmedetomidine alone

ICU - intensive care unit; WS - withdrawal syndrome; NB - newborn; GA - gestational age.

Seven RCTs ^(19-22)^ and NRCTs ^(8,23,24)^ were observed in the control and intervention groups. Five of them had weaning according to medical/conventional criteria in the control group, and the intervention group had a weaning protocol.^(8,19,22-24)^ One of them^(20)^ evaluated the control and intervention groups using dexmedetomidine. For the management and prevention of withdrawal syndrome, there was only one patient who presented was administered a placebo *versus* clonidine.^(21)^

For discussion, the studies were analyzed into two categories: “protocolized care for the prevention and treatment of withdrawal syndrome” and “use of medications for the prevention and treatment of withdrawal syndrome”.

### Protocolized care for the prevention and treatment of withdrawal syndrome

Five studies were included in this category (Table 3). Of these, three dealt with the evaluation of protocols by risk stratification in the occurrence of withdrawal syndrome^(8,22,24)^ based on the time of exposure to benzodiazepines and opioids, and one of them evaluated sedation and analgesia using scales.^(23)^ Another analyzed the occurrence of withdrawal syndrome using a medication rotation protocol.^(29)^ The most commonly used drugs for sedoanalgesia were midazolam, fentanyl and morphine, and the drugs for weaning were methadone and lorazepam.

**Table 3:** Characteristics of the protocolized care studies

Author	Protocol	Instruments	Drugs	Conversion	WS	Results
Amirnovin et al.^(8)^	Protocol for risk stratification of WS based on time of exposure to opioids and/or benzodiazepines. Low risk (< 5 days), moderate risk (≥ 5 - 7 days), high risk (> 7 to 30 days) and very high risk (> 30 days)	WAT-1	Morphine Sulfentanil Midazolam Dexmedetomidine Clonidine	Moderate risk: - Benzodiazepines → lorazepam (maximum 4mg) - Opioids **→** IV intermittent hydromorphone (0.01 to 0.06mg/kg - maximum 2mg) every 4 hours High and very high risk: oral methadone (0.05 to 0.15mg/kg - maximum 10mg) every 8 hours	4.9% *versus* 14.1%; p < 0.01	**↓** Opioid infusion (19.0 *versus* 30.0 days; p < 0.01) **↓** Benzodiazepines (5.3 *versus* 22.7 days; p < 0.01) **↓** Clonidine (14% *versus* 32%; p = 0.02) No difference in dexmedetomidine exposure
Tiacharoen et al.^(22)^	Protocol for risk stratification of developing WS, high risk: total cumulative dose of fentanyl > 0.5mg/kg; cumulative dose of midazolam > 40mg/kg; and duration of continuous intravenous infusion of opioids/sedatives > 10 days	SBS WAT-1	Fentanyl Morphine Midazolam	Fentanyl **→** oral methadone (maximum 10 mg) every 6 hours (total dose/day of fentanyl x 6.5 = dose of methadone mg/day/6 hours) Midazolam **→** oral lorazepam (maximum 2 mg) every 12 hours (total fentanyl dose/Day x 0.1 = lorazepam dose mg/day/12 hours) High risk: **↓** 10% lorazepam/methadone dose/day Low risk: **↓** 20% lorazepam/methadone dose/day	81% *versus* 84%; p = 0.865	**↓** Initial weaning phase (p = 0.026) **↓** Cumulative dose of morphine solution (p = 0.016)
Gaillard-Le Roux et al.^(23)^	Evaluation of patients with Comfort-B scale every 3 hours or NS, in order to maintain sedation levels between 11 - 17 and 7 - 11	Comfort-B SOS	Midazolam; Sulfentanil Morphine Clonidine Ketamine	Comfort-B between: (11 - 17) (< 11) (< 8) **↓** Sulfentanil 0.1µ/kg/hour **↓** Morphine 0.15 mg/kg/day Comfort-B between: (11 - 17) (8 - 11) 1-3 bolus/3 hours Comfort-B between: (> 17) (> 11) **↑**Sulfentanil 0.1µ/kg/hour **↑**Morphine 0.15 mg/kg/day	No difference	**↓** Midazolam (1 [0.56 - 1.8] *versus* 1.2 [0.85 - 2.4]; p = 0.02) No difference was shown regarding opioids
Sanchez-Pinto et al.^(24)^	Protocol for risk stratification of WS based on time of exposure to opioids and/or benzodiazepines. Low risk (< 5 days), moderate risk (≥ 5 - 7 days), high risk (> 7 to 30 days) and very high risk (> 30 days).	WAT-1	Morphine Sulfentanil Midazolam Dexmedetomidine Clonidine	Moderate risk: - Benzodiazepines **→** lorazepam (maximum 4 mg) - Opioids **→** intermittent IV hydromorphone (0.01 to 0.06mg/kg - maximum 2 mg) every 4 hours High and very high risk: - Oral methadone (0.05 to 0.15mg/kg - maximum 10mg) every 8 hours	No difference (2.6% post *versus* 4% pre; p = 0,29)	**↓** Opioid infusion (17 *versus* 22.5 days, p = 0,01) **↓** Weaning from opioids (12 *versus* 18 days, p = 0.01)
Sanavia et al.^(28)^	Medication rotation protocol. Alternating opioids with non-opioid analgesics and benzodiazepines with non-benzodiazepine sedatives.	Comfort SOS	Fentanyl Ketamine Remifentanil Midazolam Dexmedetomidine Clonidine Propofol Morphine	- First rotation (0 - 4 days): fentanyl + midazolam converted to clonidine - Second rotation (5 - 8 days): ketamine and propofol converted to metamizole - Third rotation (9 - 12 days): remifentanil and midazolam converted to clonidine - Fourth rotation (13 - 16 days): Dexmedetomidine converted to morphine	34.3% *versus* 84.6%; p < 0.001	**↓** Length of stay in the pediatric ICU (median 16 *versus* 25 days; p = 0.003) **↓** Infusion of opioids (median 5 *versus* 7 days for fentanyl; p = 0.004), benzodiazepines (median 5 *versus* 9 days; p = 0.001) and propofol (median 4 *versus* 8 days; p = 0.001) in the cohort of children in whom protocol was followed correctly

WS - withdrawal syndrome; WAT-1 - Withdrawal Assessment Tool; IV - intravenous; SBS - State Behavioral Scale; Comfort-B - Comfort-Behavior; NS - if necessary; SOS - Sophia Observation Withdrawal Symptoms Scale.

The scales used to evaluate the patients were for sedation, pain, withdrawal and *delirium*. The Withdrawal Assessment Tool 1 (WAT-1), an instrument intended for the assessment of withdrawal syndrome, was the most used and was present in three of the four studies.^(8,22,24)^

Withdrawal syndrome showed little variability between the conventional and protocol weaning groups. Two studies showed a statistically significant difference: 4.9% *versus* 14.1%, with p < 0.01,^(8)^ and 34.3% *versus* 84.6%, with p < 0.001.^(29)^

Through the application of the protocols, a reduction in the infusion of opioids was observed, as observed in four of these studies.^(8,22,24)^

### Use of medications for the prevention and treatment of withdrawal syndrome

Seven articles were included in this category (Table 4). Two dealt with the management of opioid-related withdrawal syndrome alone,^(19^.^27)^ one dealt with the use of dexmedetomidine,^(28)^ and the other four addressed the use of polytherapies with benzodiazepines and opiates.^(20,21,25,26)^

**Table 4:** Characteristics of the studies using drugs for the management and prevention of withdrawal syndrome

Identification	Infusions	Strategies	Weaning	Bolus	WS	Results
Bowens et al.^(19)^	Fentanyl infusion > 5 days; (dosages not established/NR - if patients received other therapeutic regimens)	Patients were monitored using the MNWS scale and assessed every 6 hours or NS (scores > 8 - indicative of WS). Patients were assessed 12 hours after the first dose of methadone	Low-dose methadone: 0.1mg/kg/dose (weight-based) High-dose methadone: 0.1mg/kg/dose *versus* most recent fentanyl infusion rate Methadone administered every 6 hours for the first 24 hours and every 12 hours for the next 24 hours	NR	NR	Patients who were unable complete methadone taper ↑ Fentanyl infusion time [0.58 (0.3-1.1) *versus* 0.83 (0.4-2.5) p = 0.17] **↑** Length of hospital stay [12 (9-18) *versus* 22 (16-35.3) p = 0.01]
Garisto et al.^(20)^	CONTROL 0.1mg/kg/hour midazolam, 20mcg/k/hour morphine and 7.5-15mg/kg bolus paracetamol every 6 hours D-CASE Dexmedetomidine 0.5mcg/kg/hour, midazolam 0.05mg/kg/hour, morphine 10mcg/kg/hour and paracetamol 7.5 - 15mg/kg every 6 hours	**↓** 25% sedoanalgesia if Comfort (2/2 hours) between (8 - 16) **↑** 25% sedoanalgesia if Comfort between (27 - 40) FLACC (patients with spontaneous breathing) SOS (≥ 72 hours sedoanalgesia) - evaluation for 24 hours every 8 hours	CONTROL morphine, dose of 10mcg/kg/hour D-CASE morphine 5mcg/kg/hour and dexmedetomidine 0.5mcg/kg/hour Morphine in both groups was interrupted when **↓** dexmedetomidine in 25% every 2 hours and suspended after 8 hours	At medical discretion	CONTROL *versus* D-CASE SOS 6 (IQR 3 - 8) *versus* 3 (IQR 3 - 5) - 8 horas SOS 5 (IQR 4 - 7) *versus* 3 (IQR 2 - 4) - 16 e 24 horas	**↓** Duration of MV of 33.5 (16.7-75) *versus* 41.5 (23.7-71.2) **↓** SOS scores No change in the Comfort and FLACC scales
Hünseler et al.^(21)^	Fentany 10 - 15µg/kg/hour (maximum dose) Midazolam 300 - 600µg/kg/hora (maximum dose)	Hartwing every 6 hours (having target scores of 9 - 13) Comfort Finnegan	**↓** 10 - 20% of the fentanyl and midazolam infusion every 6 hours Clonidine was initiated 96 hours after the beginning of the study, with a dose of 1µg/kg/hour and its dose was reduced by half after 48 hours of interruption of the infusion of midazolam and fentanyl	Fentanyl: 0.5 - 5.0µg/kg Midazolam: 25 - 100µg/kg Thiopental: 2 - 7mg/kg	Placebo *versus* clonidine Finnegan: 6.5 (± 2.7) *versus* 7.4 (± 2.6) (p = 0.020)	**↓** Doses of fentanyl and midazolam. Patients who used 1µg/kg/hour clonidine; **↓** signs and symptoms of withdrawal (p < 0.001)
Geven et al.^(25)^	Midazolam 0.3mg/kg/hour (maximum dose) Morphine 30µg/kg/hour (maximum dose) Dexmedetomidine 1.5µg/kg/hour (maximum dose)	Use of the Comfort scale - 3 times a day SOS - 3 times a day (≥ 72 hours sedoanalgesia) If Comfort > 17, dexmedetomidine 1.5µg/kg/hour (maximum dose) was initiated	**↓** Midazolam 0.05mg/kg/hour every 8 hours **↓** Morphine 5µg/kg/ha every 8 hours **↓** Dexmedetomidine 0.2µg/kg/hour every 8 hours	NR	Use *versus* nonuse of dexmedetomidine SOS 2 (IQR 1 - 3) *versus* 3 (IQR 1 - 4); p = 0,51	Dexmedetomidine had no preventive effect on the development of WS (p = 0.19)
Sperotto et al.^(26)^	Undefined doses (NR - if patients received other therapeutic regimens)	Evaluation of patients with Comfort-B, WAT-1 ≥ 3 and CADS ≥ 9 immediately before starting dexmedetomidine and 24 hours after dexmedetomidine infusion	NR	NR	**↓** WS (from 31/163 [19%] to 3/163 [2%]; p < 0.001) after 24 hours of dexmedetomidine infusion	**↓** Comfort-B **↓** WAT-1 *↓* CADS **↓** Dosages/kg/hour of benzodiazepines, opioids, propofol and ketamine
van der Vossen et al.^(27)^	Use of fentanyl (dosages not established)	SOS, Comfort-B and NISS applied 48 hours before replacement until 48 hours after replacement (SA if SOS ≥ 4) Comfort-B (≥ 23 or 11 - 22 with NISS of 1 = undersedation) Comfort-B score of 11 - 22 with NISS score of 2 = adequate sedation)	**↓** 10% of the initial midazolam infusion if the patient infused every 24 hours for 6 to 9 days ≥ 10 days reduction every 48 hours Oral lorazepam is calculated by dividing the daily dose of midazolam by 12, administered every 4 hours Midazolam rescue (0.1mg/kg) if SOS ≥ 4	NR	Before *versus* after use of oral lorazepam SOS scores (0 - 9) *versus* (0 - 5)	**↓** Signs and symptoms of WS **↑** Excessive sedation (**↑** NISS and Comfort-B scores) **↓** Rescue doses of midazolam and other sedatives
Berrens et al.^(29)^	Dexmedetomidine infusion for ≥ 5 days (NR - if patients received other therapeutic regimens)	A patient was considered agitated if the terms agitation, agitated or irritable were recorded in the medical records. Assessed using symptoms without a standardized scale	Weaning using clonidine: 1 - 2 days before stopping the dexmedetomidine infusion - dose of 5 to 10 mcg/kg/day Slow weaning of dexmedetomidine: weaned at 0.2µg/kg/every 6 to 12 hours	NR	NR	No difference in WS signs and symptoms between groups Clonidine did not affect the duration of dexmedetomidine weaning or length of stay in the pediatric ICU.

WS - withdrawal syndrome; NR - not reported; MNWS - Modified Narcotic Withdrawal Scale; NS - if necessary; FLACC - Face, Legs, Activity, Cry, Consolability; SOS - Sophia Observation Withdrawal Symptoms Scale; IQR - interquartile range; MV - mechanical ventilation; WAT-1 - Withdrawal Assessment Tool; CADS - Comfort-B - Comfort-Behavior; NISS - Nurse Interpretation Sedation Scale; ICU - intensive care unit.

The most commonly used drugs for sedation and analgesia were fentanyl, midazolam and morphine. Among the studies, dosages varied: midazolam varied between 0.05mg/kg/hour and 0.3mg/kg/hour, and morphine varied between 10mcg/kg/hour and 30mcg/kg/hour. Some studies considered infusion time (a minimum of 5 or more days of exposure to benzodiazepines and opioids) as an inclusion criterion.

The most commonly used scale in this category for the evaluation of withdrawal syndrome was the SOS, which was present in three of the seven studies.^(20,25,27)^ To assess sedation, the Comfort scale was the most often used (in three of the studies),^(20,21,25)^ followed by the Comfort-Behavior (Comfort-B), evaluated in two studies.^(26^.^27)^ It was also observed that one of the articles^(28)^ did not use a validated scale to observe the signs and symptoms of withdrawal, performing empirical evaluation.

Weaning varied greatly according to the protocol established by the study; however, the most commonly used drug was dexmedetomidine.

Boluses administered during opioid and benzodiazepine therapy were reported in only one study,^(20)^ making the others at greater risk of bias due to the lack of quantification of the drugs used.

Withdrawal syndrome did not show a significant reduction in incidence in the studies using drugs for weaning; only two of them showed reduced SOS scores.^(20^.^27)^

In the results, there was a reduction, especially in drugs such as midazolam and fentanyl.^(21^.^26)^

For risk assessment, RCTs were evaluated using the Revised Cochrane tool. R isk-of- B ia T hello for R andomized T rials (RoBs 2.0). The three studies classified as NRCT used Robins-I. The remaining cohorts were observational cohorts evaluated using the JBI critical evaluation checklist.^(15-18)^

The included studies generally had a high risk of bias. The RCTs had a high risk of bias regarding allocation and randomness, and the studies did not describe how this process occurred. Two of the four studies were not blinded.

The NRCTs essentially exhibited selection bias, confounding bias and intervention bias (Table 5). The observational cohort studies showed a risk of bias in items of equal measurement and exposure, free of outcome at baseline and due to losses (Table 6).

**Table 5:** Risk classification and bias of studies of randomized and non-randomized clinical trials

**Author**	**Methodology**	**Random sequence**	**Allocation confidentiality**	**Blinding of participants**	**Blinding of outcome assessors**	**Incomplete outcomes**	**Selective reporting**	**Other biases**
**RoB 2.0 Tool -RCT**								
Bowens et al.^(19)^								
Garisto et al.^(20)^								
Hünseler et al.^(21)^								
Tiacharoen et al.^(22)^								
**Robins Tool - NRCT**								
		**Bias due to confounding**	**Selection bias**	**Bias in the classification of interventions**	**Bias due to deviation of interventions**	**Bias due to missing data**	**Bias in the measurement** **of outcomes**	**Bias in the selection of results**
Amirnovin et al.^(8)^	Prospective pre- and post-intervention							
Gaillard-Le Roux et al.^(23)^	Prospective, before and after							
Sanchez-Pinto et al.^(24)^	Prospective pre- and post-intervention							

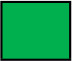
 Low risk 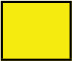 Moderate risk 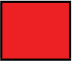 High risk 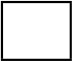 Unknown

RoB 2.0 - Revised Cochrane Risk-of-Bias Tool for Randomized Trials; RCT - randomized clinical trial; NRCT - nonrandomized clinical trials.

**Table 6:** Risk classification and bias of observational cohort studies

Author	Methodology	Similarity between groups	Equal exposure measure between groups	Valid exposure measurement	Identification of confounders	Strategies to deal with confounders	Patients free of outcome at baseline	Measure of valid results	Sufficient follow-up time	Complete follow-up/record of losses	Strategies for incomplete follow-ups were adopted	Adequate statistical analysis
Geven et al.^(25)^	Observational retrospective	N	N	Y	Y	Y	N	Y	Y	N	N	Y
Sperotto et al.^(26)^	Observational prospective	Y	N	Y	NA	NA	N	Y	N	NA	NA	Y
van der Vossen et al.^(27)^	Observational retrospective	Y	N	NA	NA	NA	N	N	Y	N	N	Y
Sanavia et al.^(28)^	Observational prospective	Y	N	Y	N	N	N	Y	Y	N	N	Y
Berrens et al.^(29)^	Retrospective observational	Y	N	Y	Y	NA	N	Y	Y	N	N	Y

N - no; Y - yes; NA - not applicable.

## DISCUSSION

This systematic review included 12 studies that determined protocols and the use of medications for the management and prevention of withdrawal syndrome. Due to the high heterogeneity in the evaluation of results and the diversity of study designs, the results were presented qualitatively, a finding similar to that of other systematic reviews that addressed this topic.^(4,7)^

Although the drugs most used for sedation and analgesia among the studies were fentanyl, midazolam and morphine, large differences in dosages were observed. The dosage of midazolam varied between 0.05mg/kg/hour and 0.3mg/kg/hour. Regarding morphine, there was also a difference between dosages, ranging from 10mcg/kg/hour to 30mcg/kg/hour. In addition, the infusion time was not noted in some studies, an important factor in determining the exposure time to the drugs.

In addition to the drugs infused, there is complexity in interpreting the results of interventions focused on the conversion of drugs for weaning because, in five studies, there was great variation regarding the drug used (clonidine *versus* placebo, methadone, dexmedetomidine, lorazepam), the route of drug administration (enteral or parenteral), time and criteria of administration. A systematic review of methadone weaning practices among pediatric intensive care patients was conducted recently, demonstrating wide heterogeneity in practices, with dosages ranging from 0.15 to 1.8mg/kg/day and dosing every 6 to 12 hours.^(30)^

The seven studies that evaluated patients using protocols of gradual reduction of sedatives and analgesics did not show significant differences regarding drug reduction. There was a single reduction in the scores of the evaluation of the withdrawal syndrome, revealing that the use of institutional protocols can demonstrate good results in terms of patient safety and optimization of resources.^(31^.^32)^ Although it is known that the use of protocols can facilitate the management of these patients, their rigid use may favor a longer duration of MV, longer stay in the pediatric ICU and greater number of reintubations. Therefore, a comprehensive view of the clinical condition of the patient and strict monitoring of pain and sedation are important. Furthermore, the protocol must clearly establish the dosages, the increase and decrease of sedoanalgesia, indications for bolus dose supplements and the method of weaning from sedation.^(32^.^33)^

According to the findings of this study, protocols and drugs for the management and prevention of withdrawal syndrome did not significantly affect its incidence, and only three studies showed a statistically significant difference.^(25,26,29)^ This fact could be attributed to the use of inadequate instruments for the study population in addition to the fact that they were not validated or translated into the language in question.

It is not enough for the instruments of health assessment to be translated into different languages; they require cultural adaptation and a specific methodology for this scale or measure to be valid in a country other than the one in which it was validated, and it must be culturally adapted to maintain its content validity in this new language and new population.^(34,35)^

However, it was observed that, among the studies that evaluated levels of sedation, four of them used the Comfort scale.^(19,20,24,29)^ However, the Comfort-B, a scale appropriate for the evaluation of sedation in children, already exists in the current literature, with important differences: the Comfort scale uses physiological variables, heart rate and blood pressure, with the intention of assessing the level of discomfort more objectively, while the Comfort-B refers only to behavioral variables, and uses an item related to crying to better assess children not on mechanical ventilation.^(22,36)^

Following the analysis, a large disparity was observed between the protocols and/or evaluation of the patients in the use of scales. SOS was used properly in only one of the studies evaluated,^(19)^ in which assessment was conducted every eight hours, or if necessary, reference scores greater than or equal to 4 were used to diagnose withdrawal syndrome, per recommendations made by the author of the scale.^(37)^

The difference between the incidence of withdrawal syndrome may be associated with the use of different protocols, drug dosages, evaluation methods and polytherapies for sedation and analgesia, factors that hinder an accurate incidence of withdrawal syndrome, thus becoming a confounding and bias-causing variable.

The use of sedoanalgesia in the treatment of critically ill children is essential, in most cases, because sedatives do not have analgesic properties, which makes their isolated use unfeasible, as they do not control pain, requiring drugs of different classes and complicating the diagnosis and treatment of withdrawal syndrome, since for each drug, there is a conduct and treatment to be performed.^(38)^

In addition, the instruments used to evaluate withdrawal syndrome, despite contemplating different signs and symptoms of withdrawal, cannot discern withdrawal syndrome caused by opioids or benzodiazepines,^(39^.^40)^ although an author^(40)^ suggests that WAT-1 is more effective in the detection of opioid withdrawal symptoms than in the detection of benzodiazepine withdrawal symptoms. This may be because, unlike the SOS, this scale does not include the specific manifestations of withdrawal from these sedatives, such as hallucinations, grimacing and disorganized movements.

One of the studies used intravenous lorazepam for the management of withdrawal syndrome, a drug that is not available in parenteral presentation in Brazil. This shows, again, the heterogeneity of the drugs used, not only for weaning but also for the management of pain and sedation in pediatric patients.

Regarding the use of drugs for the prevention and management of withdrawal syndrome, it was found that there are several protocols and drugs being studied; however, none of them have a significant impact due to the reduction of the incidence of withdrawal syndrome. Although some studies addressed the evaluation of, for example, opioids alone, this fact is not possible because most pediatric patients receive concomitant infusions of sedatives and analgesics.

Although there are studies that guide the use of medications, protocols and weaning methods for the prevention and reduction of withdrawal signs and symptoms, there is still no preestablished gold standard, and the efficacy and safety of the methods and drugs used need to be studied.

Several aspects increase the internal validity of our systematic review. First, because of the use of a search strategy based on a recognized method (PRISMA). The research was performed using the main databases available in the field of medical and health sciences. Even with great heterogeneity, the studies were classified, regardless of their methodological quality, using recommended tools, further increasing the reliability of the present study. It is believed that because withdrawal syndrome is a current topic that has received greater visibility in the past decade, the inclusion of observational studies is reasonable. Likewise, retrospective studies were included only with a sample of 50 participants or more.

This review has several limitations. Due to the small number of studies and the diversity of variables (sedoanalgesia regimen, evaluation of withdrawal syndrome, concomitant use of other drugs, patients with different pathologies and interventions), it is difficult to stratify the method or strategy most appropriate to evaluate withdrawal syndrome. Other obvious limitations are the moderate quality of the data and the limited evidence in the articles analyzed, as they include prospective and retrospective observational studies, and the NRCTs and RCTs present a significant risk of bias. This may be due to the difficulty of conducting clinical studies in the pediatric population and the scarcity of literature on the research topic. Thus, it was not possible to perform a meta-analysis due to the great heterogeneity in the methods and protocols used among the studies. Therefore, there is a need for more studies to be conducted with greater methodological rigor, including standardized protocols, with established weaning criteria, use of a homogeneous therapeutic regimen in the population and use of validated and appropriate instruments for the age group, following the guidelines suggested by the authors.

## CONCLUSION

This systematic review found great heterogeneity among the studies, especially regarding variables such as the sedoanalgesia regimen used, weaning method and evaluation of withdrawal syndrome.

Nevertheless, two studies showed a statistically significant difference in the reduction of withdrawal syndrome with the use of protocols, noting that this method may be effective for weaning from sedoanalgesia.

It is also observed that the Sophia Observation Withdrawal Symptoms Scale was the most used among the 11 studies. It is an easy-to-apply instrument that can identify the signs and symptoms of withdrawal syndrome earlier, facilitating appropriate interventions for each patient and therapeutic regimen used.

The Comfort scale was also one of the most cited instruments for assessing the levels of sedation. However, for the pediatric population, the Comfort-B scale is recommended because it allows the assessment of whether to increase or decrease sedation, which increases patient safety and reduces the effects of withdrawal syndrome.

Although it is known that there are protocols, drugs or weaning methods for the prevention and reduction of withdrawal syndrome, there is still no preestablished gold standard, and the efficacy and safety of the methods and drugs used need to be studied.

The moderate quality of the data and the scarce evidence of the articles analyzed may represent limitations because observational studies were included, which may be a consequence of the scarcity of literature on the research topic. However, all references were subjected to an evaluation of their methodological quality to identify their limitations and biases.

Further research is needed to provide more robust evidence about the most appropriate alternatives for the treatment and prevention of withdrawal signs and symptoms in critically ill children.
